# Defective Sense

**Published:** 1890-06

**Authors:** 


### DEFECTIVE SENSE.

"Prophecy" is an interior form of the ordinary faculty of "Knowledge." It is attained through an extended exercise of the ordinary senses of preception. Many men are color blind, that is, partially blind ; and the ability to see clearly, and distinguish microscopically or telescopically, varies greatly, and may be educated like any other sense. Those who live in a narrow area of bricks and mortar, and stare at books nearly all the time, become short-sighted ; whereas the sailor, and traveller in open country, can see for great distances, and trace sound inaudible to untrained ears. As in col r blindness, a curious instance of imperfect hearing is furnished in the case of Mr. Edwin Cowles, editor and owner of the *Cleveland Leader*, one of the greatest newspapers of the West. Until Mr. Cowles was twenty-five years old, he supposed that all he heard and read about the songs of birds was poetical fiction, since to his ears they had always been as mute as fishes. Then a distinguished aurist, learning of this droll delusion, sought Mr. Cowles out, and made the discovery that he could not hear the notes of a piano or organ above the sixth octave, or even the shrillest or most vibrant high notes of a fife or violin. Put in a room with twenty canary birds, Mr. Cowles could not hear the slightest sound, even when they were singing at their shrillest, and he placed his ear close to the cage. All the sibilant sounds in human speech escaped him likewise, and as a result he never produced them in his own talk. Yet, strange enough, in all other respects, his hearing was more than ordinarily acute."

